# Matrix metalloproteinases (MMPs) family gene polymorphisms and the risk of multiple sclerosis: systematic review and meta-analysis

**DOI:** 10.1186/s12883-020-01804-2

**Published:** 2020-05-29

**Authors:** Mina Mohammadhosayni, Arezou Khosrojerdi, Keivan Lorian, Saeed Aslani, Danyal Imani, Bahman Razi, Farhad Babaie, Shahram Torkamandi

**Affiliations:** 1 Department of Immunology, Faculty of Medicine, Shahed University, Tehran, Iran; 2 Department of Medical Immunology, Faculty of Medical Sciences, Tarbiat Modares University, Tehran, Iran; 3 Department of Physiology, School of Medicine, Tehran University of Medical Sciences (TUMS), Tehran, Iran; 4 Department of Immunology, School of Medicine, Tehran University of Medical Sciences, Tehran, Iran; 5 Department of Immunology, School of Public Health, Tehran University of Medical Sciences, Tehran, Iran; 6 Department of Hematology and Blood Transfusion, School of Medicine, Tarbiat Modares University, Tehran, Iran; 7 Cellular and Molecular Research Center, Urmia University of Medical Sciences, Urmia, Iran; 8 Department of Medical Genetics and Immunology, Faculty of Medicine, Urmia University of Medical Sciences, Urmia, Iran

**Keywords:** Multiple sclerosis, Central nervous system, Matrix metalloproteinases, Genetic polymorphism, Meta-analysis

## Abstract

**Background:**

Several studies have reported the association between polymorphisms in Matrix metalloproteinases (MMPs) gene family and risk of Multiple sclerosis (MS). However, the results have been inconsistent and inconclusive. To resolve this issue, here we performed a systematic review and meta-analysis of the MMP-91562 C/T (rs3918242), MMP-3 (− 1612 5A/6A), and MMP-2 (− 1306 C/T) polymorphisms and susceptibility to MS.

**Methods:**

We conducted a comprehensive systematic search in the major electronic database, including Scopus and PubMed to look up for relevant studies published before December 2019 that surveyed the association between the MMP-91562 C/T (rs3918242), MMP-3 (− 1612 5A/6A), and MMP-2 (− 1306 C/T) polymorphisms and susceptibility to MS. The level of association between the polymorphisms and susceptibility to MS in the polled analysis was determined by calculating the odds ratio (OR) and the corresponding 95% confidence interval (CI).

**Results:**

We found 15 studies containing 2430 MS subjects and 2304 controls. A statistically significant association was observed in the all five comparisons of the MMP-91562 C/T polymorphism and MS risk as follows: dominant model (OR = 1.62, 95% CI = 1.03–2.53, *P* = 0.03), recessive model (OR = 2.69, 95% CI = 1.68–4.29, *P* < 0.001), allelic model (OR = 1.51, 95% CI = 1–2.28, *P* = 0.04), TT vs. CC model (OR = 3.20, 95% CI = 1.87–5.46, *P* < 0.001), and CT vs. CC model (OR = 1.53, 95% CI = 1.02–2.28, *P* = 0.04).

**Conclusions:**

Our meta-analysis revealed significant association of MMP-9 (− 1562 C/T) Single-nucleotide polymorphism (SNP) with MS susceptibility that increased the disease risk.

## Background

Multiple Sclerosis (MS) is a chronic autoimmune disease of the brain and spinal cord of the central nervous system (CNS) that is characterized by demyelination, inflammation and axonal degeneration, resulting in serious disability in young adults [1, 2]. It is estimated that above 2.5 million people in the world suffer from MS [3]. Although the main etiology of the disease remains obscure, it is thought that both genetic and environmental factors and their interactions are critical in disease development [4–6]. Especially, the role of genetic factors in the pathogenesis of MS has been established by family and twin studies. Accordingly, the heritability of MS is estimated to be about 25–80% [7–9]. As noted in recent studies, several genes, including interleukin (IL) 6, IL-12, vitamin D receptor (VDR), Signal transducer and activator of transcription (STAT) 4, Protein tyrosine phosphatase, non-receptor type 22 (PTPN22), CD40, programmed cell death (PD1/PD-L1), and Matrix metalloproteinases (MMPs) have been correlated with MS and attracted much attention to investigating more genetic factors contributing to MS risk [10–17].

MMPs are zinc-dependent endopeptidases enzymes that play an important role in many physiological and pathological processes including inflammation, invasiveness of tumor, metastasis, and angiogenesis by the degradation of the extracellular matrix and basement membrane (BM) [18, 19]. On the basis of structure and in terms of substrate specificity, MMPs are divided into six groups: collagenases (MMP-1, − 8, − 13, − 18), gelatinases (MMP-2, − 9), stromelysins (MMP-3, − 10, − 11), matrilysins (MMP-7, − 26), membrane-type MMPs (MMP-14, − 15, − 16, − 17,-24, − 25) and other non-classified MMPs (MMP-12, − 19, − 20, − 21, − 22, − 23, − 27, − 28) that is encoded by a separate gene and has a different tissue distribution and bioactive function [20–22]. MMPs have a critical role in the pathogenesis of MS by inducing migration of immune cells through the blood-brain barrier (BBB) into the CNS, which seems essential during the formation of inflammatory lesions [23].

The regulation of MMP family genes expression has not been understood, but it has been demonstrated that genomic sequence of MMP family genes is polymorphic; it is of added attention to ascertain which polymorphisms in MMP family genes have functional potentials to influence the final bioavailability of family member(s) and therefore the progression of MS [14, 24–26]. Several studies have evaluated the association between MMP family gene polymorphisms and MS risk; but the results are often inconsistent [27–29]. Therefore, we performed a meta-analysis to attain a consistent conclusion of the association between the MMP-91562 C/T (rs3918242), MMP-3 (− 1612 5A/6A), and MMP-2 (− 1306 C/T) gene polymorphisms and susceptibility to MS.

## Methods

This meta-analysis was performed by sticking to the Preferred Reporting Items for Systematic Reviews and Meta-Analyses (PRISMA) statement [30]. The current meta-analysis does not contain any studies with human participants or animals performed by any of the authors.

### Search strategy

The initial comprehensive and systematic search was conducted in Medline, Scopus, and PubMed databases. To be assured of our search, combination of following key words and Medical Subject Headings (Mesh) terms were used: (“matrix metalloproteinase” [Mesh] OR “MMP” OR “gelatinase”) AND (“multiple sclerosis” OR “MS”) AND (“single nucleotide polymorphism” OR “SNP” OR “polymorphisms” OR “mutation” OR “variation”). We retrieved all studies published prior to January 2020. The references of reviews and eligible studies were cross-checked to prevent missing of any eligible study which was not identified by primary search.

### Inclusion and exclusion criteria

Studies considered eligible if met the following criteria: 1) Publications evaluating the association between MMP family gene polymorphism and susceptibility to MS as main outcome; 2) Publications with case and control groups (Case-control design and cohort design); 3) Publications which report odds ratio (OR) and 95% confidence interval (CI) or crud data to calculate these items; 4) Publications with sufficient data such as genotype distribution and allele frequency. Publications like reviews, meta-analysis, case reports, book chapters, letter to editors, conference abstracts, as well as duplicates were all excluded.

### Data extraction and quality assessment

We identified eligible studies by sticking to the inclusion and exclusion criteria, and to perform meta-analysis following data were extracted: the first author’s name, journal and year of publication, country of origin, ethnicity, number of subjects in the case and the control groups for each gender, mean or range of age, genotyping method, genotype counts in the case and control groups. It is noteworthy that all procedure of data extraction was performed by two authors independently and possible discrepancy was solved by consensus. Furthermore, the quality of each study was assessed using the Newcastle-Ottawa Scale (NOS) criteria [31]. Studies with scores 0–3, 4–6 or 7–9 were of low, moderate, or high-quality, respectively.

### Statistical analysis

We used Pearson’s chi-square test in control groups to estimate Hardy–Weinberg equilibrium(HWE) for each study. In this study OR with 95% CI was used to assess the strength of the association between MMP family gene polymorphism and MS risk. The genotype model which defined for MMP-2, MMP-3, and MMP-9 were as follow: **MMP-2** (Dominant model [TT + CT vs. CC], Recessive model [TT vs. CT + CC], Allelic model [T vs. C], Homozygote contrast [TT vs. CC], and Heterozygote contrast [CT vs. CC]); **MMP-3** (Dominant model [6A6A + 5A6A vs. 5A5A], Recessive model [6A6A vs. 5A6A + 5A5A], Allelic model [6A vs. 5A], Homozygote contrast [6A6A vs. 5A5A], and Heterozygote contrast [5A6A vs. 5A5A]); **MMP-9** (Dominant model [TT + CT vs. CC], Recessive model [TT vs. CT + CC], Allelic model [T vs. C], Homozygote contrast [TT vs. CC], and Heterozygote contrast [CT vs. CC]). Possible heterogeneity in this study was estimated by Q-test and *I*^*2*^ test [32, 33]. Accordingly, a *P* value< 0.10 of *Q*-test and *I*^*2*^ < 50% demonstrate no evidence of heterogeneity and fixed effect model (FEM) was used [34]. But, if *P* value> 0.10 for *Q*-test and *I*^*2*^ > 50%, then the study was considered heterogeneous and random effect model (REM) was applied [35]. Furthermore, publication bias was measured by Egger’s regression test, Begg’s adjusted rank correlation test, and visual examination of the funnel plot (*P* value< 0.05 was considered statistically significant) [36]. Finally, we performed sensitivity analysis to observe the impact of any individual study on the pooled OR. Statistically analyses were carried out using STATA (version 14.0; Stata Corporation, College Station, TX) and SPSS (version 23.0; SPSS, Inc. Chicago, IL).

## Results

### Study selection

**Fig. 1:**
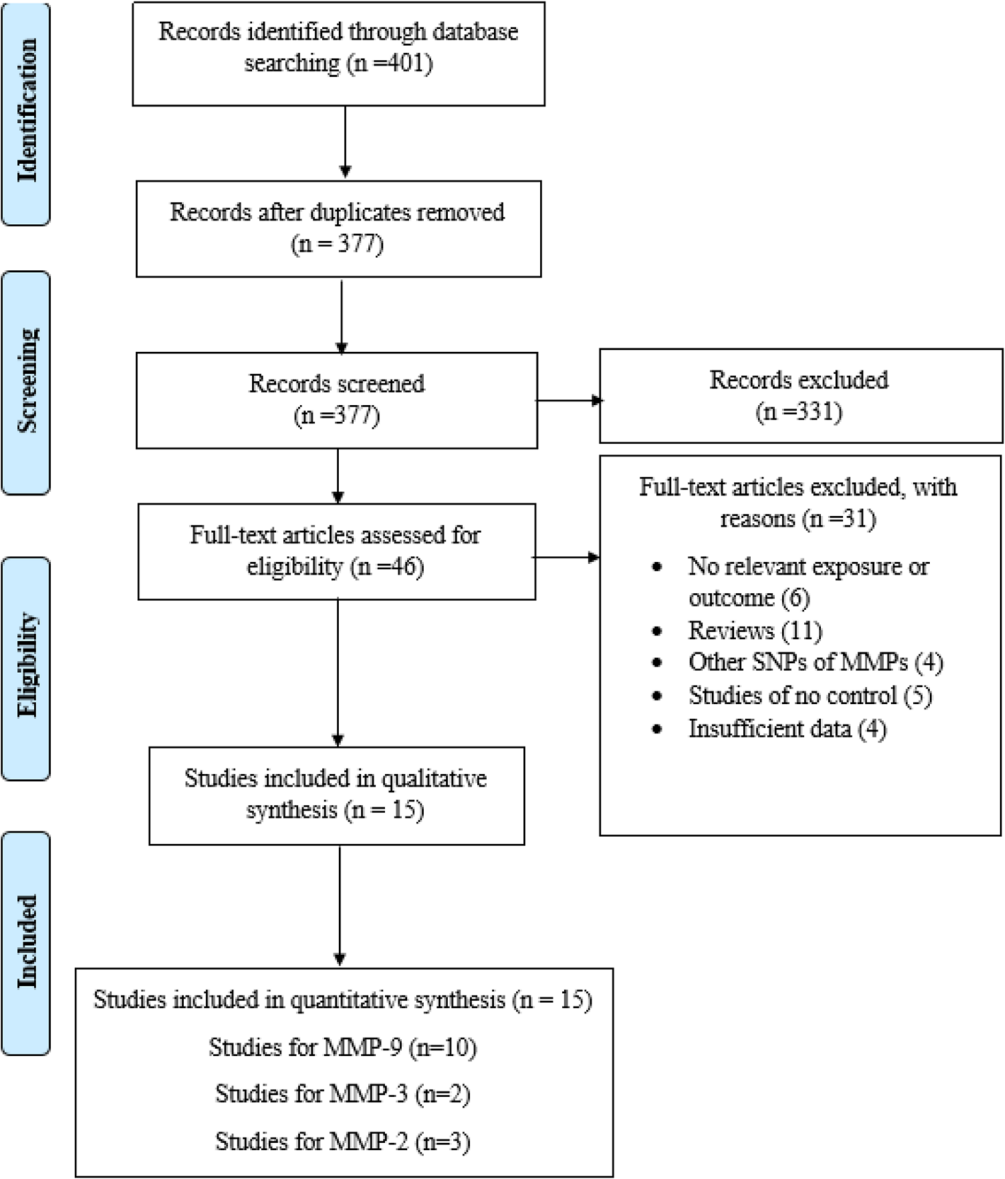
Flow diagram of study selection process

Based on aforementioned key words, primary search generated 401 studies that 24 studies were duplicates. The other 377 studies were screened according to the inclusion and exclusion criteria. Taken to gather, 331 studies were excluded by title and abstract screening and 31 studies were excluded after full text examination. Ultimately, 15 studies matched with the inclusion criteria and selected for quantitative analysis. Tables 1 and 2 summarize the characteristics and genotype frequency of the included studies. The mean age of case and control groups were between 30 and 40. All studies had good methodological score ranging from 5 to 8 and were published between 2000 to 2019. Among included studies, the Restriction fragment length polymorphism (RFLP)-PCR was used as a common genotyping model. The screening workflow and study selection process are shown in Fig. 1.

**Table 1:** Characteristics of studies included in meta-analysis

Study author	Year	Country	Ethnicity	Total cases/controls	Sex cases/controls	Genotyping method	Mean age Cases/Controls	Quality score
**MMP9**								
Nelissen et al.	2000	Sweden	European	199/146	M = NR F=NR	PCR-RFLP	NR/NR	6
Zivkovic et al.	2007	Serbia	European	187/282	M = 67/140 F = 120/142	Touch Down PCR	35.5 ± 10.1/40.8 ± 14.8	7
Benesova et al.	2008	Czech Republic	European	244/132	M = 63/45 F = 181/87	PCR-SSP	38.4 ± 10.2/35.6 ± 11.7	7
Mirowska-Guzel et al.	2009	Poland	European	234/190	M = 66/76 F = 168/114	PCR-RFLP	40.09 ± 10.19/40.09 ± 10.19	7
Fernandes et al.	2009	Brazil	South-American	158/191	M = 41/54 F = 117/137	PCR-RFLP	38.7 ± 13/35.6 ± 9.5	6
La Russa et al.	2010	Italy	European	243/173	M = 96/107 F = 147/66	PCR-RFLP	41.1 ± 12.2/28.5 ± 9.4	7
Valado et al.	2017	Portugal	European	169/183	M = 48/63 F = 121/120	PCR-RFLP	41.44 ± 0.84/39.09 ± 0.96	6
Ibrahim et al.	2019	Egypt	African	50/100	M = 18/NR F = 32/NR	PCR-RFLP	32.9 ± 8.1/NR	5
Sabbagh et al.	2019	Iran	Asian	100/105	M = 37/41 F = 63/64	PCR-ARMS	42.89 ± 10.48/46.52 ± 8.90	5
Sadr et al.	2019	Iran	Asian	170/200	M = 121/142 F = 49/58	PCR-RFLP	33.34 ± 7.91/31.88 ± 9.79	7
**MMP3**								
Djuric et al.	2008	Serbia	European	184/236	M = NR F=NR	Touch Down PCR	NR/NR	7
Rahimi et al.	2016	Iran	Asian	121/106	M = 24/17 F = 97/89	PCR-RFLP	35.3 ± 9.1/34.5 ± 11.4	6
**MMP2**								
Benesova et al.	2008	Czech Republic	European	240/132	M = 60/45 F = 180/87	PCR-SSP	38.4 ± 10.2/35.6 ± 11.7	7
Aksoy et al.	2016	Turkey	European	102/102	M = 76/75 F = 26/27	Taq man	36.69 ± 8.33/35.93 ± 8.20	6
Liutkeviciene et al.	2018	Lithuania	European	26/26	M = NR F=NR	Taq man	36/34	5

**Table 2:** Distribution of genotype and allele frequencies among MS patients and controls

Study author	MS cases					Healthy control					P-HWE	MAF
	CC	CT	TT	C	T	CC	CT	TT	C	T		
**MMP9**												
Nelissen et al.	143	51	5	337	61	102	40	4	244	48	0/973	0/164
Zivkovic et al.	146	41	0	333	41	200	74	8	474	90	0/716	0/16
Benesova et al.	191	50	3	432	56	87	42	3	216	48	0/424	0/182
Mirowska-Guzel et al.	128	103	3	359	109	136	50	4	322	58	0/811	0/153
Fernandes et al.	117	35	6	269	47	156	32	3	344	38	0/369	0/099
La Russa et al.	164	73	6	401	85	147	25	1	319	27	0/954	0/078
Valado et al.	130	35	4	295	43	145	34	4	324	42	0/247	0/115
Ibrahim et al.	28	21	1	77	23	78	21	1	177	23	0/751	0/115
Sabbagh et al.	11	35	54	57	143	42	42	21	126	84	0/087	0/4
Sadr et al.	96	60	14	252	88	163	33	4	359	41	0/144	0/103
**MMP2**												
Benesova et al.	143	84	13	370	110	75	40	17	190	74	0.004	0.28
Aksoy et al.	40	56	6	136	68	77	25	0	179	25	0.15	0.123
Liutkeviciene et al.	19	7	0	45	7	190	108	20	488	148	0.38	0.233
**Study author**	**MS cases**					**Healthy control**					**P-HWE**	**MAF**
	**5A/5A**	**5A/6A**	**6A/6A**	**5A**	**6A**	**5A/5A**	**5A/6A**	**6A/6A**	**5A**	**6A**		
**MMP3**												
Djuric et al.	24	102	58	150	218	37	130	69	204	268	0.06	0.568
Rahimi et al.	3	66	52	72	170	1	67	38	69	143	0.006	0.675

P-HWE, *p*-value for Hardy–Weinberg equilibrium; *MAF* Minor allele frequency of control group

### *Meta-analysis of MMP-9 (*− 1562 C/T*) and risk of MS*

**Fig. 2:**
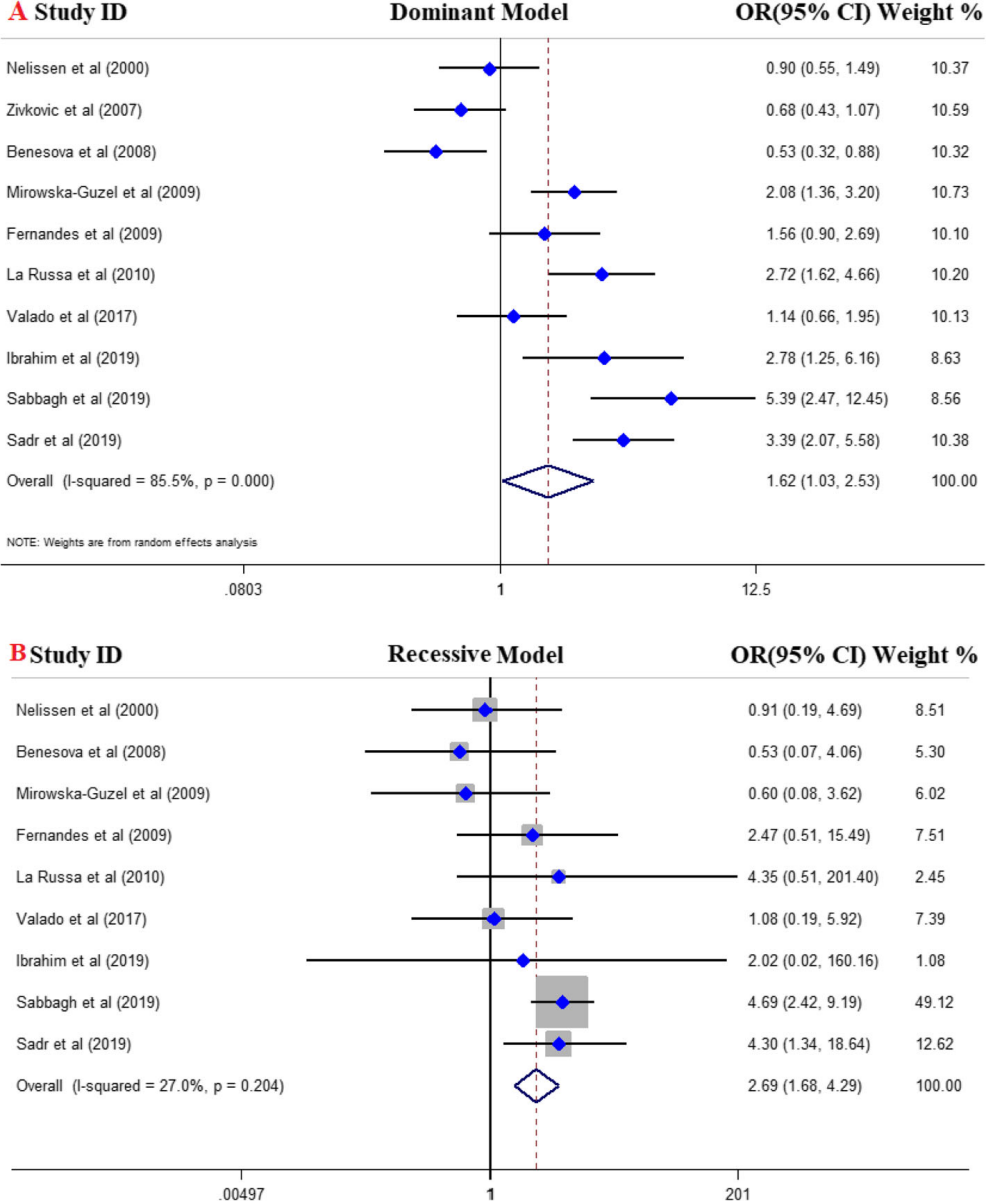
Forest plot of the association between *MMP-9* gene polymorphism and MS risk: A; dominant model, B; recessive model

A total of 10 eligible studies with 1754 cases and 1702 controls were included in quantitative analysis. Of them, six studies were conducted in European countries [27, 29, 37–40], two studies in Asian countries [41, 42], one study was in Egypt [43] and one in Brazil [44]. The pooled OR divulged a strong positive association between *MMP-9* gene rs34016235 polymorphism and risk of MS and announced this SNP as a risk factor for MS (Fig. 2). In details, dominant model (OR = 1.62, 95% CI = 1.03–2.53, *P* = 0.03), recessive model (OR = 2.69, 95% CI = 1.68–4.29, *P* < 0.001), allelic model (OR = 1.51, 95% CI = 1–2.28, *P* = 0.04), TT vs. CC model (OR = 3.20, 95% CI = 1.87–5.46, *P* < 0.001), and CT vs. CC model (OR = 1.53, 95% CI = 1.02–2.28, *P* = 0.04). FEM was used for recessive and homozygote compressions and REM was applied for dominant, heterozygote, and allelic models. The results of pooled ORs, heterogeneity tests and publication bias tests in different analysis models are shown in Table 3.

**Table 3:** Main results of pooled ORs in meta-analysis of *MMP* family gene polymorphism

Subgroup		Sample size	Test of association		Test of heterogeneity		Test of publication bias (Begg’s test)		Test of publication bias (Egger’s test)	
	Genetic model	Case/Control	OR	95%CI(*P*-value)	*I2* (%)	P	Z	P	T	P
**MMP9**										
**Overall**	Dominant	1754 / 1702	**1.62**	**1.03– 2.53 (0.03)**	85.5	≤0.001	0.80	0.42	1.27	0.24
	Recessive	1754 / 1702	**2.69**	**1.68 – 4.29 (≤0.001)**	27	0.20	-1.46	0.14	-2.17	0.06
	Allelic	1754 / 1702	**1.51**	**1- 2.28 (0.04)**	87.8	≤0.001	0.27	0.78	0.34	0.74
	TT vs. CC	1754 / 1702	**3.20**	**1.87 – 5.46 (≤0.001)**	50	0.04	-1.04	0.29	-1.90	0.9
	CT vs.CC	1754 / 1702	**1.53**	**1.02 – 2.28 (0.04)**	80.8	≤0.001	0.98	0.32	1	0.34
**MMP3**										
	Dominant	305/342	1.18	0.66-2.13 (0.57)	0	0.57	-1	0.31	*	*
	Recessive	305/342	0.57	0.18-1.79 (0.33)	88.5	≤0.001	1	0.31	*	*
	Allelic	305/342	1.11	0.88-1.41 (0.39)	0	0.91	1	0.31	*	*
	6A6A vs. 5A5A	305/342	1.15	0.62- 2.11 (0.43)	0	0.48	-1	0.31	*	*
	5A6A vs.5A5A	305/342	1.24	0.64 – 2.39 (0.52)	0	0.54	1	0.31	*	*
**MMP2**										
**Overall**	Dominant	368 /552	1.36	0.39 – 4.78 (0.07)	90.4	≤0.001	0.52	0.60	0.04	0.97
	Allelic	368 /552	1.15	0.36 – 3.61 (0.81)	91.9	≤0.001	0.52	0.60	0.14	0.91
	CT vs.CC	368 /552	1.50	0.52 – 4.35 (0.45)	85.9	≤0.001	0.52	0.60	-0.10	0.93

* Egger’s test was not calculable

### Meta-analysis of MMP-3 (− 1612 5A/6A) and risk of MS

Two studies with 305 cases and 342 controls were included. One study was conducted in Serbia [45] and the other in Iran [46]. The results of overall population reject any association between *MMP-3* gene *− 1612 5A/6A* SNP and risk of MS across all genotype models including; dominant model (OR = 1.18, 95% CI = 0.66–2.13, *P* = 0.57), recessive model (OR = 0.57, 95% CI = 0.18–1.79, *P* = 0.33), allelic model (OR = 1.11, 95% CI = 0.88–1.41, *P* = 0.39), 6A6A vs. 5A5A model (OR = 1.15, 95% CI = 0.62–2.11, *P* = 0.66), and 5A6A vs. 5A5A model (OR = 1.24, 95% CI = 0.64–2.39, *P* = 0.52). The results of pooled ORs, heterogeneity tests and publication bias tests in different analysis models are shown in Table 3.

### *Meta-analysis of MMP-2 (*− 1306 C/T) *and risk of MS*

Overall, three studies with 368 cases and 552 controls were eligible for the association between *MMP-2* gene rs243865 SNP and susceptibility to MS. All three studies were conducted in Europe [38, 47, 48]. Because of TT genotype frequency of zero in both cases and controls, the recessive model and TT vs. CC model were not applicable to calculate. The results of other three models also reject association between *MMP-2* gene rs243865 SNP and MS risk. The results were; dominant model (OR = 1.36, 95% CI = 0.39–4.78, *P* = 0.07), allelic model (OR = 1.15, 95% CI = 0.36–3.61, *P* = 0.81), and CT vs. CC model (OR = 1.50, 95% CI = 0.52–4.35, *P* = 0.45). The results of pooled ORs, heterogeneity tests and publication bias tests in different analysis models are shown in Table 3.

### Publication bias and heterogeneity

**Fig. 3:**
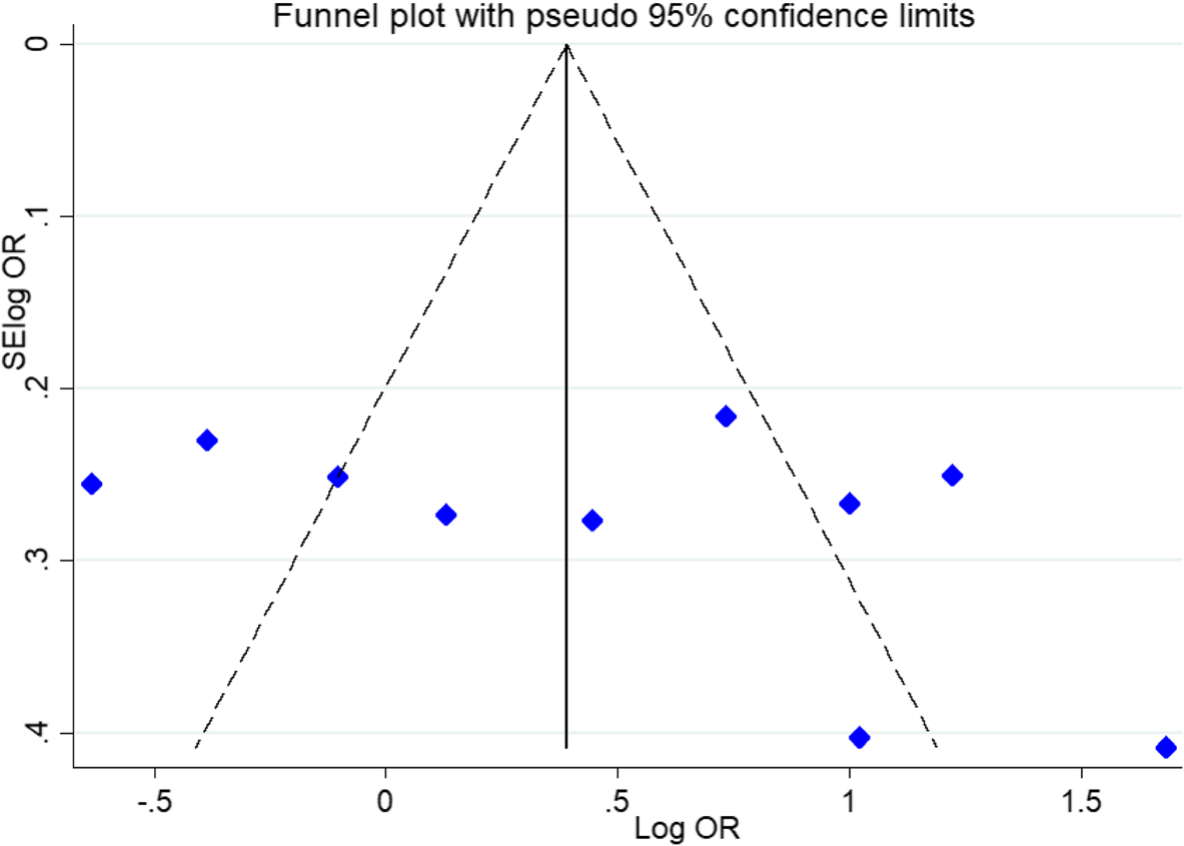
Begg’s funnel plot for publication bias test. Each point represents a separate study for the indicated association

In this study, we used Egger’s regression test, Begg’s adjusted rank correlation test and visual examination of the funnel plot to measure publication bias. The results of Begg’s and Egger’s tests for *MMP-9*1562 C/T (rs3918242), *MMP-3* (− 1612 5A/6A), and *MMP-2* (− 1306 C/T) gene polymorphisms showed no evidence of publication bias (Fig. 3). During our analysis, we have detected some degree of heterogeneity for three SNPs (Table 3).

### Sensitivity analysis

**Fig. 4:**
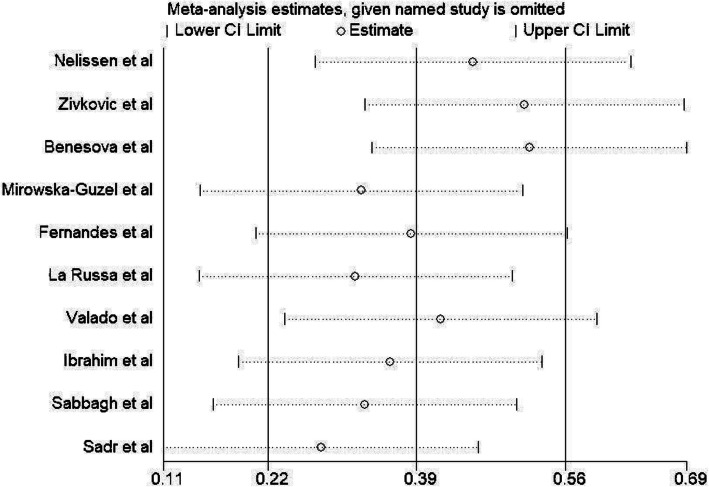
Sensitivity analysis in present meta-analysis investigates the individual influence of studies on pooled results

Here, we performed sensitivity analysis only for *MMP-9* gene rs3918242 SNP in order to determine whether sequential omission of the eligible studies affect the final pooled OR. As shown in (Fig. 4), the result was not changed, confirming the stability of our meta-analytical result.

## Discussion

To date, several surveys have been conducted addressing the possible association between polymorphisms of the MMP gene family, including *MMP-9*1562 C/T (rs3918242), *MMP-3* (− 1612 5A/6A), and *MMP-2* (− 1306 C/T) and risk of MS, resulting in inconsistent and inconclusive results. Through resolving the limitation of insufficient statistical power and small sample size in individual studies, meta-analysis studies confer a beneficial approach to settle the problem of conflict. In order to meet this need, here in this meta-analysis we included the most comprehensive and up-to-date original works to obtain exact approximation with respect to association between MMP gene polymorphisms and MS risk.

Previously, large genome-wide association study (GWAS) did not reveal that MMP9 polymorphisms to be significantly associated with the risk for MS [49]. Additionally, a meta-analysis performed on the case-control studies from Europe and one Brazilian study and did not indicate a significant association of MMP9 (− 1562 C/T) SNP with and MS susceptibility [50]. The number of included studies in the meta-analysis was limited and inclusion of further recent studies in Asian populations, like Iran and Egypt, led to significant association of MPP9 polymorphism with MS susceptibility. On the other hand, the role of variations in the MMP9 gene may differ in various populations. Reports have indicated that the T allele was underrepresented only in female MS patients [37, 38]. It seems that differences in the genetic originality of subjects in different populations as well as the gender of the MS patients are involved in determining the function of MMP gene variations in the etiopathogenesis of MS.

MMPs are enzymes that have proteolytic function and have been attributed with numerous implications in tissue remodeling and development. MMPs, especially MMP-9 (also known as gelatinase B), have been observed to be the primary enzymes involved in the degradation of the BBB in MS setting [23, 51]. The major function of MMP-9 is to degrade extracellular matrix (ECM) and myelin basic protein (MBP), hence mediating the recruitment of the inflammatory cells into the involved CNS in MS disease [52–55]. MS patients have shown increased cerebrospinal fluid (CSF) and serum levels of MMP-9 [56, 57]. MMP-9 level has been implied to be a proper marker for the evaluation of clinical type and severity of the disability in MS patients [58].

Studies have established that two functional SNPs in the promoter region MMP-9 gene, namely rs3918242 and rs3222264, impress the expression of this gene [39, 44, 59, 60]. In rs3222264 SNP, the − 90 position is involved in double strand DNA opening and exerted by transcription factors and DNA regulatory proteins. In vitro experiments indicated that the C–1562 T SNP play a role in blocking the nuclear repressor protein binding to the promoter region in which this SNP is located, leading to upregulation of MMP-9 expression [61].

Our meta-analysis indicated significant association of MMP-9 (− 1562 C/T) SNP and risk of MS. Interestingly, all genetic model comparisons, including dominant model (OR = 1.62), recessive model (OR = 2.69), allelic model (OR = 1.51), TT vs. CC model (OR = 3.20), and CT vs. CC model (OR = 1.53), increased the risk of MS susceptibility. The previous meta-analysis by Li et al. in 2017 included 6 studies comprising 1265 MS patients and 1104 controls. The meta-analysis did not show any association of MMP-9 (− 1562 C/T) SNP with MS risk [50]. However, our meta-analysis, by including 10 studies for MMP-9 (− 1562 C/T) SNP containing 1757 MS subjects and 1702 controls, indicated an increased risk of MS by all genetic models of this polymorphism. Furthermore, we evaluated the possible association of *MMP-3* (− 1612 5A/6A) and *MMP-2* (− 1306 C/T) SNPs and risk of MS to attain more comprehensive conclusion of MMP gene polymorphisms and MS risk. However, we did not find association of these SNPs with MS risk, possibly due to small sample size and little number of studies, which need further evaluations in the future.

Although *MMP-3* (− 1612 5A/6A) and *MMP-2* (− 1306 C/T) SNPs were not associated with MS risk according to our meta-analysis, the role of genetic interactions and haplotypes should not be neglected. In a study, the MMP-9 T allele was not associate with MS risk, however, a synergism was identified between MMP-9 C and MMP-7 G alleles in increasing MS risk by 1.5 times. Furthermore, there was 3.13 times increased MS risk in association with the haplotype MMP-9 T, MMP-7 G, and MMP-2 C (TGC) in comparison to the CAG haplotype [28]. Therefore, further studies may disclose the association on *MMP-3* (− 1612 5A/6A) and *MMP-2* (− 1306 C/T) SNPs with MS risk in the haplotype analysis.

Although we tried to carry out the most comprehensive meta-analysis of the MMP gene SNPs and the risk of MS, a number of limitations of this meta-analysis study should be remarked. First, the number of studies and sample size for *MMP-3* (− 1612 5A/6A) and *MMP-2* (− 1306 C/T) SNPs in this meta-analysis was relatively small to conclude the role of these SNPs and MS risk. Second, we only searched for articles published in the English language and a number of potential data might be neglected. Third, the current meta-analysis was according to crude analysis of the genetic polymorphisms, and the adjusting the analysis by gender, age, and other environmental factors were not taken into consideration. Fourth, we detected some degrees of heterogeneity for the analyzed SNPs, that might stem from difference in genetic stratification and ethnicity, variety in the environmental factors in different populations, and the detection methods.

## Conclusion

Taken all the evidence into conclusion, this was the first and most comprehensive evaluation of the MMP gene family SNPs in association with MS. Unlike previous meta-analysis, our study detected significant association of MMP-9 (− 1562 C/T) SNP with increased risk of MS. Nonetheless, other polymorphisms were not associated, perhaps due to little sample size. Hence, we acknowledge the further studies with respect to evaluation of other MMP gene SNPs in association with MS, particularly in a haplotype analysis. Furthermore, the role of other factors, like age, gender, and environmental factors in the analysis ahead will hopefully shed further light on the bona fide implication of MMP gene polymorphisms and susceptibility to MS.

## Data Availability

All data that support the conclusions of this manuscript are included within the article.

## Abbreviations

MMP: Matrix metallo proteinases
MS: Multiple sclerosis
CNS: Central nervous system
CI: Confidence interval
VDR: Vitamin D receptor
STAT: Signal transducer and activator of transcription
PTPN22: Protein tyrosine phosphatase, non-receptor type 22
OR: Odds ratio
SNP: Single-nucleotide polymorphism
PRISMA: Preferred Reporting Items for Systematic reviews and Meta-Analyses
NOS: Newcastle–Ottawa scale
HWE: Hardy–Weinberg equilibrium
